# Mapping local knowledge supports science and stewardship

**DOI:** 10.1007/s13280-025-02170-4

**Published:** 2025-04-26

**Authors:** Sarah C. Risley, Melissa L. Britsch, Joshua S. Stoll, Heather M. Leslie

**Affiliations:** 1 School of Marine Sciences, University of Maine, 227 Libby Hall, Orono, ME 04469 USA; 2 University of Maine Ecology and Environmental Sciences Program, Orono, ME 04469 USA; 3 University of Maine Darling Marine Center, 193 Clarks Cove Rd, Walpole, ME 04573 USA; 4 Maine Coastal Program, Maine Department of Marine Resources, 21 State House Station, Augusta, ME 04333 USA

**Keywords:** Fisheries management, Local knowledge mapping, Maine, Participatory mapping, Shellfish fisheries, Social–ecological systems

## Abstract

Coastal marine social–ecological systems are experiencing rapid change. Yet, many coastal communities are challenged by incomplete data to inform collaborative research and stewardship. We investigated the role of participatory mapping of local knowledge in addressing these challenges. We used participatory mapping and semi-structured interviews to document local knowledge in two focal social–ecological systems in Maine, USA. By co-producing fine-scale characterizations of coastal marine social–ecological systems, highlighting local questions and needs, and generating locally relevant hypotheses on system change, our research demonstrates how participatory mapping and local knowledge can enhance decision-making capacity in collaborative research and stewardship. The results of this study directly informed a collaborative research project to document changes in multiple shellfish species, shellfish predators, and shellfish harvester behavior and other human activities. This research demonstrates that local knowledge can be a keystone component of collaborative social–ecological systems research and community-lead environmental stewardship.

**Supplementary Information:**

The online version contains supplementary material available at 10.1007/s13280-025-02170-4.

## Introduction

Understanding and managing in the face of the complexity of coastal marine social–ecological systems (SESs) require fine-scale information that accurately represents the socioeconomic and ecological dynamics of the system (Ban et al. 2009; Rowell et al. 2022). Yet, this resolution of information is not always available. Limited fine-scale data can contribute to an incomplete understanding of the social and ecological components of a system, inadequate indicators and time scales for research and monitoring, or conservation actions and analyses that do not reflect the rate of change of the SES (Guerrero et al. 2013). Local-scale decision-making, or other bottom-up environmental management strategies, is restricted to a small geographic area and thus requires fine-scale information salient at this spatial scale (Bellwood et al. 2019; Falco et al. 2022). In contrast, geographically more extensive decision-making processes, often led by state, provincial, or federal government agencies, may not have the same need for fine-scale information. Yet there are many examples of when fine-scale information highlights how to effectively tailor environmental policies to heterogeneous social–ecological contexts (e.g., Alessa et al. 2008; Leslie et al. 2015).

In the Gulf of Maine, USA, stewardship challenges in coastal marine SESs are compounded by rapidly warming waters and changes in coastal environments (Pershing et al. 2021). Maine’s shellfish fishery has experienced changes over the last decades, including a decline in soft-shell clam populations (Tan and Beal 2015), a decrease in access to harvest areas due to pollution closures of mudflats following increasingly intensive rainfall events (Evans et al. 2016), and diminishing physical access points to the intertidal due to coastal development (Britsch 2022), in addition to a reemergence of wild oyster populations in regions where they were once functionally extinct (Capone et al. 2008). These rapidly shifting conditions in Maine’s coastal marine SESs have highlighted the need for fine-scale, local data on how these systems and their small-scale fisheries are changing through time. Unfortunately, natural resource managers and local leaders working in these spaces have limited data to support decision-making. This is particularly true of Maine’s shellfish fishery: (1) monitoring the intertidal mudflat environments inhabited by shellfish is challenging, time-consuming, and costly, and (2) state and local governing bodies responsible for stewarding wild shellfish populations often lack the capacity to comprehensively study these environments.

The shellfish fishery in Maine is managed through a system of co-management (Title 12, Part 9, Chapter 623), in which the Maine Department of Marine Resources and coastal towns share responsibility for maintaining the health of commercially important shellfish. Coastal towns can establish municipal shellfish ordinances and shellfish committees, which can charge license fees, establish areas open or closed to harvest, and create harvesting limits. While a small number of coastal towns have sufficient capacity and data to support local decision-making about shellfish resources, most would benefit from additional activities that build both capacity and knowledge (McGreavy et al. 2018).

In the past decade, coastal marine researchers, managers, and resource users have identified local knowledge as an integral part of the sustainable stewardship of marine resources and ecosystems (Thornton and Scheer 2012; Campbell et al. 2024). We follow Berkes and colleagues’ definition, which describes local knowledge as the place-based, experiential development of a relevant body or system of knowledge by the people who live, work, and depend upon an ecosystem (Berkes et al. 2000). Unlike prevailing approaches to coastal marine monitoring and management that focus primarily on ecological dimensions of a system—such as the dynamics of a single species or environmental variable—local knowledge is not siloed into a single category. Rather, local knowledge communicates connections in systems and spans social and ecological dimensions (Tengö et al. 2021).

Participatory approaches to research and stewardship can support shellfish co-management, as this governing structure encourages collaboration and co-production of knowledge (Berkes 2009). These research approaches focus on building collaborative projects from the bottom-up by engaging with communities to generate information and develop practical solutions to community-identified problems (Brown et al. 2022). This shift in perspective has highlighted the capacities of communities to develop solutions for problems identified locally (Kumar 2002). In this collaborative research model, researchers function primarily as facilitators in the research process, supporting local participation and equitable exchange between researchers and community collaborators. Bottom-up approaches like this do come with their own challenges—including a lack of community buy-in or diversity in participation, persistent power structures that prioritizes the knowledge of ‘elite actors’ over others, as well as the difficulty in maintaining sustainable, long-term partnerships among diverse actors (Moallemi et al. 2023)—but still present a promising alternative way forward.

Participatory mapping is an important methodology for documenting local knowledge to fill data gaps and inform decision-making in coastal marine SESs (Karnad 2022). As an overarching term, ‘participatory mapping’ describes diverse approaches that solicit local knowledge to document, analyze, and represent various forms of spatial data, often in the form of maps (Schmitz Nunes et al. 2021). Participatory mapping in a fisheries management context often occurs as a co-productive process in which researchers and managers rely upon fishers’ knowledge to provide information about a system (Leite and Gasalla 2013). In practice, participatory mapping has played an essential role in assessing data-poor species (Beaudreau and Levin 2014); informing the design of marine-protected areas (Mellado et al. 2014); and characterizing broader relational connections or "sense of place" among people and the environments in which they live and work (Brown et al. 2020; Campbell et al. 2024). By fostering collaborative engagement between researchers, managers, and coastal communities, participatory mapping is uniquely positioned to lay the foundation for collaborative, locally relevant, and action-oriented research (Walter 2009).

Local knowledge has proved instrumental in marine research and stewardship. Local knowledge has helped refine the focus of research projects, by informing the design of manipulative experiments (Bart 2006) or by providing initial assessments of the study system (Arce-Ibarra and Charles 2008). Traditional ecological knowledge, with a specific focus on indigenous knowledge, has also been used to identify specific locations or ecosystem dynamics that require further inquiry (Drew 2005). Local knowledge also has informed the design and management of marine-protected areas (e.g., Ban et al. 2009).

Although previous participatory mapping studies have supported aspects of research in coastal marine SESs, rarely have the results directly informed the design and development of collaborative research projects in the Northeastern US. Established management practices in North America and Europe rely heavily on conventional scientific methods (NOAA 2021; Beier et al. 2024), although there are outstanding examples from Brazil, New Zealand, and Australia where participatory mapping and related approaches has been foregrounded (Brown et al. 2020; Borges et al. 2021; Auliagisni et al. 2022). Researchers have emphasized the need to include local knowledge and participatory approaches in US fisheries research and management (e.g., Wilson et al. 2018), but in practice these approaches have been infrequently applied within the Northeastern USA (see Ames 2003; Nenadovic et al. 2012; Sutton 2020; Hart et al. 2022). Few studies are designed to develop place-based, collaborative, and adaptive research and stewardship practices that address local questions.

Here we report on the role of participatory mapping of local knowledge in the context of designing collaborative research projects in rapidly changing coastal marine SESs. We show how beginning with participatory mapping helps to: (1) generate fine-scale, spatially explicit data informing the "where?" of collaborative research, (2) provide valuable historical and contextual information informing the ‘how?’ of collaborative research, (3) capture social and ecological data in tandem, informing the "what?" of collaborative research, and (4) frame and characterize the "why" of collaborative research. We report the results of this process from two focal SESs in the Damariscotta and Medomak River estuaries in Maine, USA. Ultimately, this research serves as an example for how participatory mapping can be foundational to the development of research projects in rapidly changing coastal marine SESs.

## Theoretical framework

This research is guided by the social–ecological system (SES) framework (Ostrom 2009). We use this theoretical framework to identify and refine our hypotheses and to guide empirical work in coastal marine SESs. We employed an SES framework tailored specifically for benthic small-scale fisheries to inform our approach to knowledge accumulation and synthesis of social and ecological variables and processes (Basurto et al. 2013). Overall, our work takes a participatory and pragmatic research approach with the goal of shared inquiry and knowledge co-creation between the researchers and research partners (Guba and Lincoln 2005; Moon and Blackman 2014). Working within this research paradigm, we understand that the goal of knowledge generation is action and transformation and have designed our methods so that the knowledge documented through this work can be applied to local-level decision-making and scientific inquiry. We recognize that knowledge is experiential and that new knowledge can be generated through its application and through democratic dialogue among researchers and partners (Guba and Lincoln 2005). As researchers, we acknowledge that our personal beliefs and identities directly impact how we relate to and interpret our work. We strive to communicate our positionalities with our partners and provide opportunities in our methods for reflexivity on how our personal standpoints impact our results (Erickson 2018). Our research looks toward examples of community science for design and structure and most closely aligns with the "Community engages with external science organizations" typology described by Charles et al. (2020). However, the project is nuanced and can also be classified as the "Community engages with resident scientists" model because the project’s lead researcher is a member of the shellfish committee and the University researchers contributing to the project often live and work on the Damariscotta. Therefore, the project can be best classified as a resident scientist model nested within the model of external science organizations (Charles et al. 2020).

## Materials and methods

### Focal social–ecological systems

**Fig. 1:**
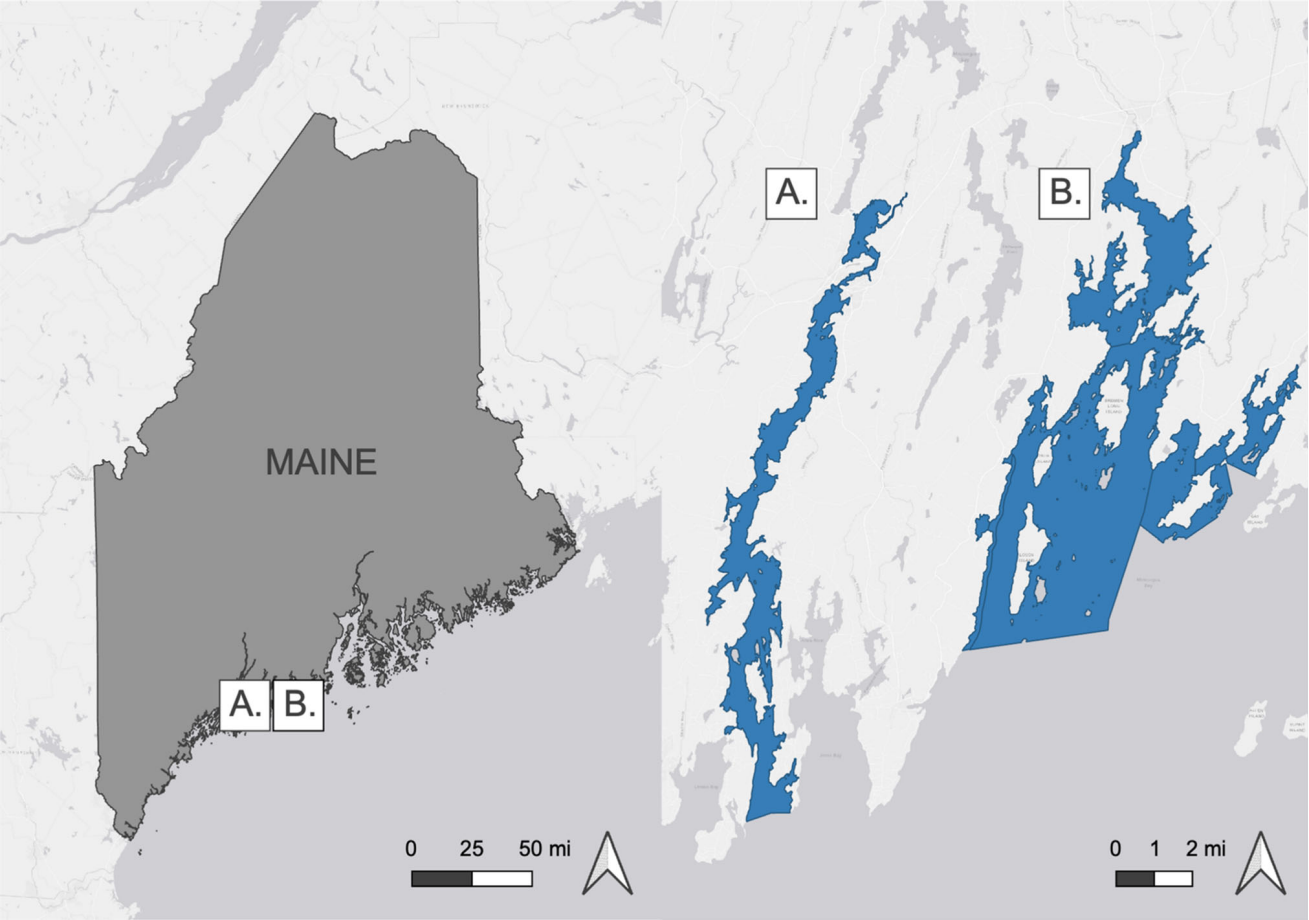
Locations of the two focal SESs in Maine, USA. **A** Damariscotta River; **B** Medomak River

We conducted this study in two focal SESs associated with wild shellfish fisheries in the Damariscotta River and the Medomak River estuaries (Fig. 1). Shellfish committee members from both focal systems expressed interest in our assistance. Their questions and concerns inspired our research efforts. Both SESs have diverse coastal marine habitats, including rocky shore and soft sediment intertidal areas, subtidal eelgrass beds and kelp forests, and subtidal rocky and soft sediment benthic environments. The Damariscotta is home to more than a dozen marine aquaculture operations totaling ~ 1 km^2^, where farmers grow bivalve shellfish and kelp species (DMR 2023). In contrast, the Medomak has only two active aquaculture farms, which together occupy 0.03 km^2^ (DMR 2023). The intertidal area of the upper Medomak River estuary, managed by the Towns of Bremen and Waldoboro, has an active shellfish fishery involving more than 200 harvesters. In contrast, the Damariscotta’s shellfish fishery, managed by the Towns of Damariscotta and Newcastle, involves fewer than 10 active shellfish harvesters within the upper estuary. Maine’s shellfish fishery is primarily focused on soft-shell clams (*Mya arenaria*), but quahogs (*Mercenaria mercenaria*), razor clams (*Ensis directus*), and more recently, wild American oysters (*Crassostrea virginica*, hereafter "wild oysters") are also harvested. Both SESs support other human activities, including lobster (*Homarus americanus*) and Atlantic menhaden (*Brevoortia tyrannus*) fisheries, and recreational boating and fishing.

This study was the first step in the development of a collaborative research effort between the towns of Damariscotta and Newcastle and our research group. Additional details about how the community science project in the Damariscotta River has developed can be found in Risley et al. (2023).

### Participatory mapping of local knowledge

We collaborated with 50 local shellfish harvesters and other community members (whom we refer to as "general users" in the results reported below), applying participatory mapping to document local knowledge of shellfish populations, areas of human use, and locations of importance in the Damariscotta River and Medomak River focal SESs. Participatory mapping generates geospatial data through a collaborative mapping process with local knowledge holders (Schmitz Nunes et al. 2021). In adherence to ethical guidelines, all research was approved by the University of Maine’s Institutional Review Board (IRB application #2020-06-16).

**Fig. 2:**
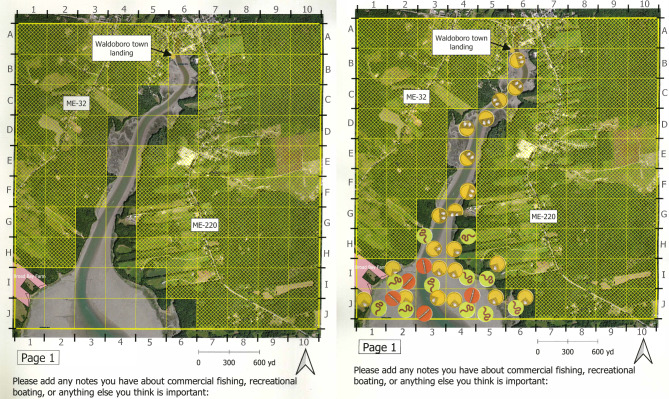
Example of an uncompleted and a completed map page. The map on the left shows a map page prior to completion, with grid cells onto which stickers may be placed. The map on the right was completed by a participating shellfish harvester

We solicited input from municipal leaders, shellfish resource managers, and academic researchers to inform our study design, including selection of a base map (Fig. 2) and identification of the types of information to be collected from different types of participants (Table 1) (Close and Hall 2006). We chose aerial photographs that show important landscape features and intertidal mudflat areas for the base map based on expert recommendations. Important landmarks (e.g., town landings and road names) were added to further orient study participants. We overlaid the maps with a grid (Fig. 2) and designed stickers that participants would place on the map to show the locations of shellfish species and human activities (Table 1). We made these design choices because study participants completed the maps independently during the COVID-19 pandemic. Grids streamlined the data collection and entry process (de Oliveira Leis et al. 2019), while stickers helped to engage participants and standardize map responses.

**Table 1:** Map stickers for the two different types of study participants: the general user of the estuary vs. the shellfish harvester. Each participant received only one version of the study (sticker diameter: 1.27 cm)

General user		Shellfish harvester	
Sticker image	Description	Sticker image	Description
	Aquaculture		Soft-shell clams—abundance low
	Recreational fishing		Soft-shell clams—abundance medium
	Sailing		Soft-shell clams—abundance high
	Tourism and sightseeing		Razor clams
	Kayaking		Quahog/hard clams
	Area of significant change		Wild oysters
			Marine worms
			Area of significant change

We identified study participants based on their experience and activity in the focal SESs. All participants needed to have active experience within the last 1–3 years and were asked to only complete map pages for areas in the focal SES where they had recent, direct knowledge. Although some participants may have had experience across both SESs, each participant was only sent the version of study for the system with which he/she was most familiar (e.g., a shellfish harvester who has commercial fishing licenses for both SESs but spends most of their time on the Medomak would only complete the study for that SES). Maps were intended to represent a snapshot of participant observations within recent memory, which we defined as the past 1–3 years. We divided participants into two groups—(1) general user and (2) shellfish harvester—based on whether they were a general user of the estuary (described in more detail below) or were a shellfish harvester (i.e., held a commercial or recreational shellfishing license). We identified participants using lists of town recreational and commercial shellfish licenses, state commercial shellfish, lobster, and worm harvesting licenses, and our prior knowledge of people involved in the aquaculture industry, environmental conservation, and waterfront businesses. We confirmed our initial participant groupings during recruitment by asking individuals to describe different ways that they engage with the study system.

General user participants in the Damariscotta system self-identified as individuals involved in an array of activities: recreation on the estuary (*n* = 15), tourism (*n* = 3), municipal or state management (*n* = 6), aquaculture (*n* = 3), research or conservation (*n* = 7), and lobster fishing (*n* = 1). Note that some participants belong to multiple sub-categories. Nine of the shellfish harvester participants held a commercial shellfish license, while three held a recreational license. In the Medomak, general user participants self-identified as individuals involved in recreation on the estuary (*n* = 9), aquaculture (*n* = 3), municipal management (*n* = 2), research or conservation (*n* = 1), or other marine industries (*n* = 1). All shellfish harvester participants held commercial shellfish licenses (Table 2). Although some participants’ experiences span broad categories, each participant was only asked about the activity (general use vs. shellfish harvesting) which they self-identified as being most familiar. We identified additional participants via snowball sampling, whereby individuals are identified based on the recommendations of past participants (Creswell 2014).

**Table 2:** Demographic information of study participants

Focal system	Study type	Participants (No.)	Male	Female	Median age	Avg. Yrs. experience
Medomak	General user	14	8	6	53	25
Medomak	Shellfish harvester	8	8	0	59	32
Damariscotta	General user	17	14	3	57	31
Damariscotta	Shellfish harvester	11	10	1	55	35

Our sampling design was uneven due to demographics of the human populations associated with the focal SESs, as well as recruitment challenges perhaps unique to the shellfish harvesting community (Table 2). We contacted and attempted to recruit all licensed shellfish harvesters across both focal SESs (> 150 individuals). However, individuals working in marine industries, including shellfish harvesting, have time- and labor-intensive occupations, making participation in research difficult. As a result, our sample may not fully represent the broader population of harvesters in these estuaries. Additionally, we had few female participants (Table 2). Marine industries in the Northeastern US tend to be male dominated, leading to a lower number of females available for recruitment. These factors contributed to an uneven sampling design, despite our efforts to reach a diverse group of participants.

We recruited participants over the phone and by email by making, at minimum, three attempts to contact. Table 2 shows the demographics of participants for both focal SESs. After receiving consent, we mailed "mapping packets" to each participant’s home address. Mapping packets consisted of a large envelope containing a coil-bound map booklet consisting of zoomed in map pages (dimensions 27.9 cm × 38.1 cm), sticker sheets corresponding to the participant’s expertise (General User or Shellfish Harvester), a zoomed-out guide map showing the exact location of each map booklet page within the estuary (dimensions 45.7 cm × 76.2 cm), writing utensils, and detailed instructions. Because of the commercial significance of soft-shell clams as the primary species of interest for shellfish committees, we provided abundance stickers for users to indicate high, medium, or low abundance within the estuary. All other species of interest to shellfish committees (razor clams, quahogs, wild oysters, and marine worms) and human use activities (sailing, kayaking, recreational fishing, aquaculture, and tourism/sightseeing) had stickers indicating presence (Table 1). A final sticker category, "Areas of Significant Change," could be placed to show locations where participants identified or observed important change within the past 1–3 years (Table 1). Individual participants defined "Areas of Significant Change" based on their personal experiences. Participants placed the stickers on the maps and wrote additional comments as notes within the map booklet (de Oliveira Leis et al. 2019). Participants returned their completed maps in self-addressed envelopes that were included in their mapping packet. Participatory mapping took place between October 2020 and January 2021.

### Semi-structured interviews

We scheduled interviews with participants at the time of recruitment. Interviews occurred after participants completed the mapping packets. The interviews were semi-structured and focused on open-ended questions about participant observations of change in the focal SESs within the past 1–3 years. The interviews provided qualitative context for the mapping data and allowed participants to describe their experiences and participant observations in their own words (Creswell 2014). We conducted 50 interviews between October 2020 and January 2021. Four participants completed the mapping packet but declined to interview, while three participants interviewed but did not complete the mapping packet. Given the time that participants invested in these activities and that the "incompleteness" of several respondents’ materials did not impact our analytical approach or results, we chose to report all data. Interviews were approximately 30 to 90 min in length, were recorded, and occurred over the phone or via video conference due to COVID-19 precautions.

### Analysis

We digitized the map booklets by assigning a unique number identifier to each grid cell and manually entering the information from the map booklets as binary data into a spreadsheet. For example, if a participant placed stickers for quahogs, oysters, and soft-shell clams within grid #288, we placed a ‘1’ in each of these three categories for row #288. We captured any handwritten comments as notes for the specified grid cells. All aggregated map data follow the US standard for the "fisheries rule of three" (although standards vary internationally), as defined by the National Oceanic and Atmospheric Administration (NOAA), to maintain confidentiality (NOAA 1994). That is, occurrences that were observed by three or more participants for a particular grid were included in the aggregated maps.

We imported the non-confidential map data into R (Version 1.2.0553) as CSV files to create raster map layers. We created multiple types of maps: (1) presence–absence maps with "filled" and "unfilled" grid cells, (2) abundance maps with distinct categories (high, medium, and low), and (3) heat maps with gradients, showing data like human activity. Soft-shell clam abundance (high, medium, and low) was self-reported by participants by completing the following statement as part of the mapping activity: “Thinking about the last 2–3 years, to me high numbers of clams means I can harvest X bushels of soft-shell clams in a tide.” Although responses varied among participants and not all participants provided their own definitions, low abundance was defined as ½—1 bushel of soft-shell clams harvested in a low tide, mid-abundance as 1–2 bushels, and high abundance 2–3+ bushels (*n* = 5). We also overlaid map layers with existing layers, like the Maine Department of Marine Resources Aquaculture Lease Map (DMR 2023), to examine overlapping human activities in the focal SESs.

With each participant’s permission, we audio recorded and then transcribed interviews verbatim using a two-part process. First, we used an artificial intelligence software, Otter.ai, to automatically transcribe interviews. Next, we manually reviewed each transcript to check for transcription errors, for example, the misinterpretation of local terminology or location names that were unfamiliar to the AI software. Once interviews were transcribed, we analyzed them using the qualitative data analysis software NVivo R1 in two cycles.

To begin, we honed our research concerns based on a literature review and questions raised by fisheries managers during prior research (Creswell 2014). We used this review to develop a preliminary coding framework grounded in our research interest in characterizing long-term social and ecological change within the systems. We then looked to the interview text to develop a complete list of themes. Our coding practice begins with recognizing patterns and themes within the raw text, developing emergent themes based on repeated ideas expressed in the text, and then connecting these themes to higher level theory and research concerns of our study (Auerbach and Silverstein 2003). We as a research team then cross checked these emergent themes and developed a synthesized list of themes. In total, the research team identified 21 upper-level themes ranging in topics from "Tourism" to "Water Quality" (see supplemental material for complete schema). Nine high-level themes related to participant observations of change. For each observed change, we next coded potential drivers of change based on participant observations of what factors may be causing change.

We also used interview data to characterize the magnitude and direction of change. For each participant who mentioned a change, we analyzed the context and applied a value to characterize the direction of the change. For example, on the theme of "Aquaculture Activity," Participant A may observe, “Aquaculture has increased dramatically in the last two years,” while Participant B may say, “Aquaculture hasn’t really grown in our estuary yet.” In this example, Participant A has described an increase in aquaculture activity, characterized as a + 1, while Participant B has observed no growth in aquaculture activity, characterized as a value of 0. We then aggregated the values of each characterization to quantify the magnitude (as defined by the number of participants who observed the change) and direction (increase, decrease, or no change) of changes observed by our participants.

## Results

### Fine-scale and spatially explicit information

**Fig. 3:**
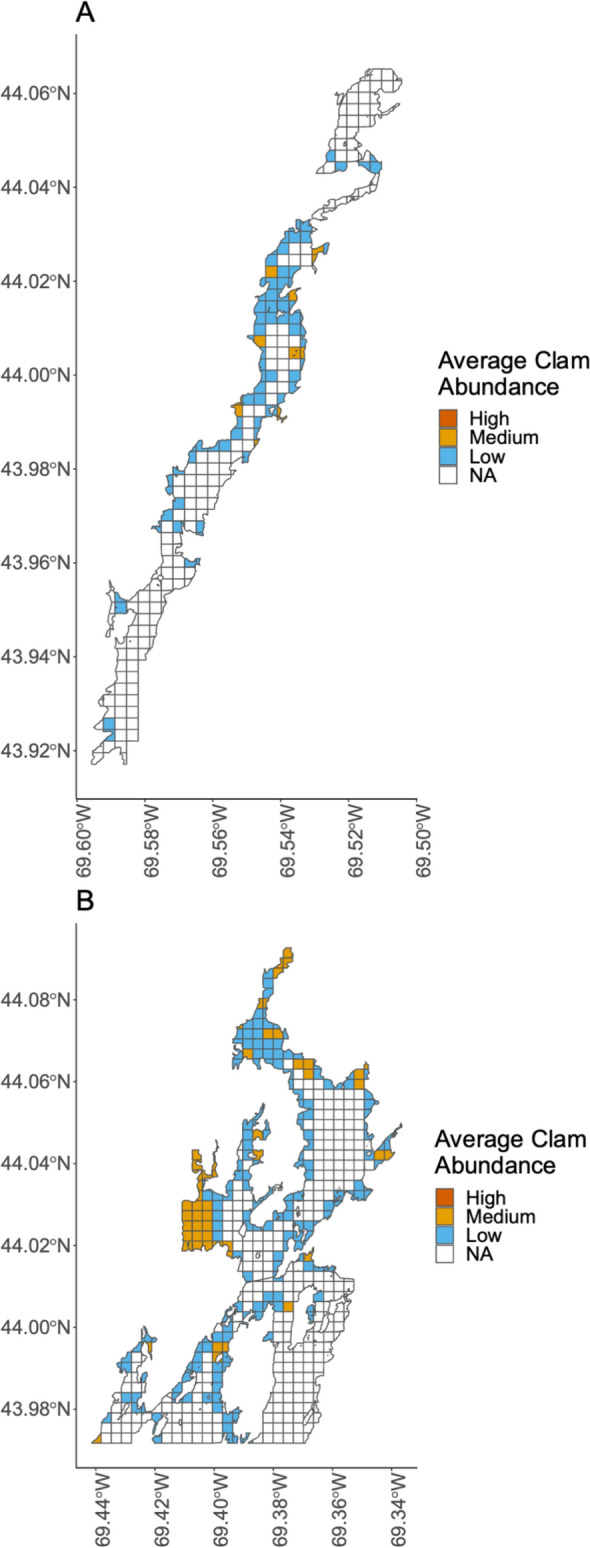
Average soft-shell clam abundance in the two focal SESs. **A** Participants (*n* = 11) identified areas with high, medium, and low soft-shell clam abundance in the Damariscotta River. **B** Participants (*n* = 7) identified areas with high, medium, and low soft-shell clam abundance in the Medomak River. Although participants reported areas of high abundance, average responses resulted in only medium or low abundances

The maps we co-produced with harvesters through this participatory mapping study provided fine-scale spatial information on soft-shell clam abundance and distribution in the Damariscotta River and Medomak River focal SESs (Fig. 3). The maps were a snapshot of conditions in the systems within the past 1–3 years. These maps showed important harvesting locations of high clam abundance and locations of low clam abundance that may benefit from restoration activities, like re-seeding.

**Fig. 4:**
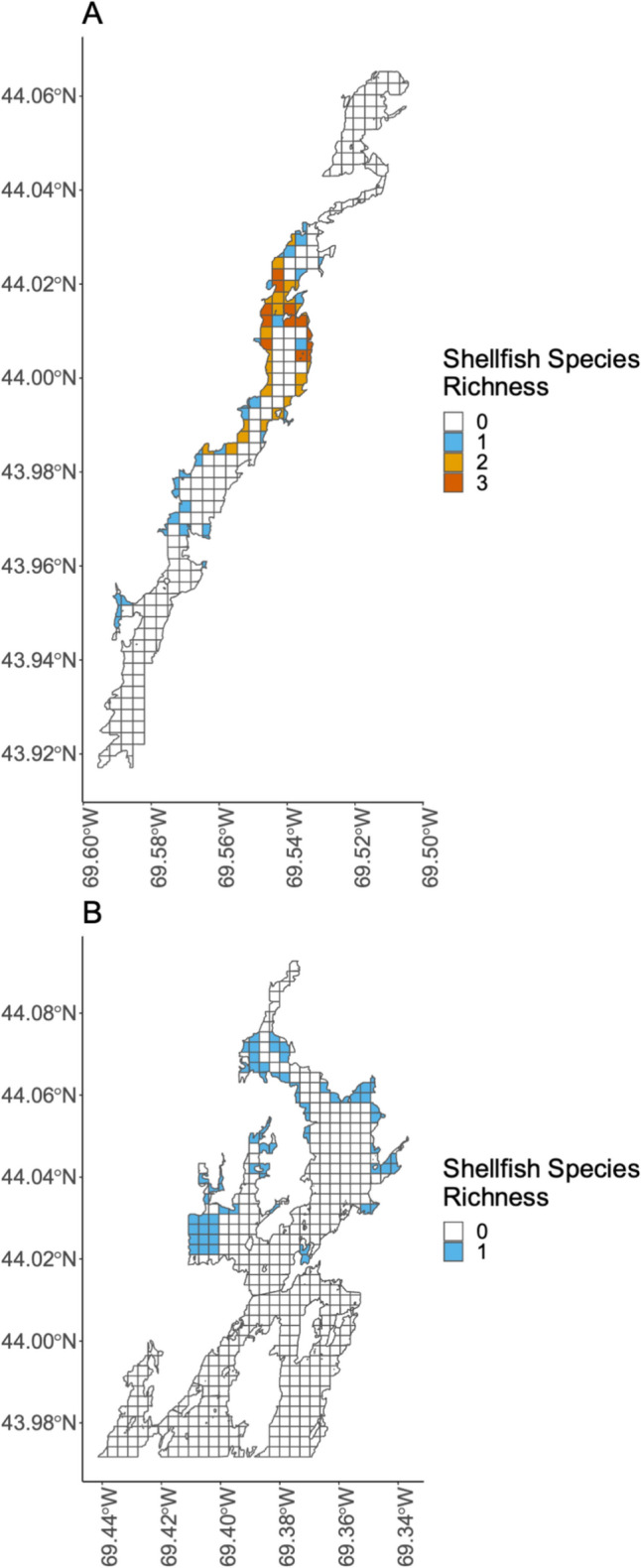
Shellfish species richness in the two focal SESs: **A** Damariscotta River; **B** Medomak River. Richness is the total number of shellfish species observed by study participants, including soft-shell clams, wild oysters, razor clams, and quahogs

We also generated maps that characterized species richness of shellfish in the two focal SESs. Medomak shellfish harvester participants reported a shellfish richness of 1 or fewer for all intertidal areas, indicating that only one shellfish species is present, while Damariscotta participants reported a shellfish richness of 1–3 for intertidal areas, indicating a greater shellfish species richness in the Damariscotta than in the Medomak (Fig. 4).

### Multiple methods address multiple time scales

While local knowledge maps represented a snapshot in time, interview results provided decadal understandings of change. Although recent history (1–3 years) was the primary focus for both the interviews and maps, participants often reflected on changes spanning much longer timescales during the interviews. Many reported perceptions of change that dated back to the beginning of their experience in the systems.

Participants from both focal SESs described perceived changes in the abundance and species richness of commercially important shellfish species through time. For example, Medomak participants witnessed declines in razor clam populations. As one shellfish harvester shared, “There used to be razor clams in the Medomak'' while another reported, “…I don't think there’s as many as it used to be.” Additionally, shellfish harvesters perceived a rise in quahog populations—“…there’s more quahogs I’ve ever seen since I was a kid” or “There’s some quahogs around now…I mean we get some guys that come in that are finding them down there on the big tides…harvesting 3000 or 4000 to a tide.” Participants also reported observations of wild oysters, a previously extirpated species that has experienced a population resurgence (Larsen 2016; Britsch 2021). One Damariscotta participant noted this increase in wild oysters relative to other shellfish abundance:

“…the wild oyster population took off…in the last 40 years, we’ve…seen a definite decrease in the species of razor clams and soft-shell clams and an increase in oysters and [quahogs].”

Participants also observed how shellfish habitat and species-specific distributions have shifted. For example, four participants described how soft-shell clam habitat once encompassed the large area of soft, fine sediment mudflats stretching from the mid to the low intertidal zone. Over time, this band narrowed. In the past harvesters, “…could go way out in the mud, but now there’s no clams out there.” Now they harvest, “say 25–30 feet from the high-water mark down” observed one Damariscotta participant. This was echoed in the Medomak: “…all these clams that used to be all the way to the channel marker, now there’s almost zero anywhere, they’re all on the shore.”

Participants characterized the changing distribution of shellfish species in both focal SESs (Table 3). Our interview results indicated that soft-shell clams are less abundant in the soft, fine-grained sediments (i.e., mud) of the mid- and low intertidal zone, and more abundant in the mid to high intertidal zones, where sand, gravel, rocks, and coarse-grained sediments predominate (Table 3).

**Table 3:** Shellfish habitat and intertidal distribution information in the Damariscotta (*n* = 11) and Medomak (*n* = 7) River focal SESs, based on interview data. ‘NA’ refers to the cases where fewer than three participants shared observations, or where these species were not commonly found in the focal SES

Focal SES	Species	Habitat	Tidal distribution
Damariscotta	Soft-shell clam	Soft mud to sandy, rocky areas	Upper to low intertidal zone
	Quahog	Sandy, rocky shore, lives closer to the surface than soft-shell clams	Mid to low intertidal zone
	Razor clam	Sandy areas or softer mud	Low intertidal, almost subtidal
	Wild oyster	Attached to rock or other substrates, often under seaweed or on rocky, shell, and gravel areas of shore	Upper intertidal zone or low intertidal zone
Medomak	Soft-shell clam	Soft mud to hard mud, clay, sandy, shelly, rocky areas	Mid to upper intertidal zone
	Quahog	Harder mud, sand, or gravel areas	NA
	Razor clam	Soft mud to shelly, rocky mud	NA
	American oyster	NA	NA

**Fig. 5:**
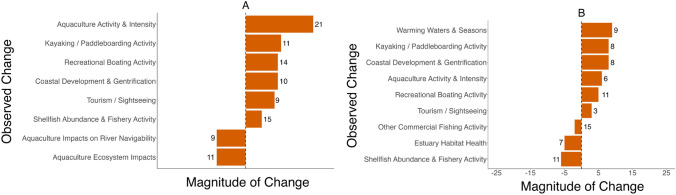
Changes observed by study participants in the focal SES: **A** Damariscotta River (*n* = 28); **B** Medomak River (*n* = 22). Bars show the observed magnitude of the change and whether the perceived change was an increase (+) or a decrease (−). The number at the end of each bar shows the total number of participants who observed each change and contributed to the net value shown. Changes with a value of zero illustrate that participants held diverse opinions about an observed change and indicate that both an increase and a decrease were simultaneously observed by participants

The decadal scale information from local knowledge interviews also provided perceptions of large-scale changes in the focal systems. We documented participant perceptions of change and the number of individuals that identified changes as significant to understand the perceived magnitude of changes in the systems (Fig. 5). Our results indicate an increase in aquaculture activity is one of the commonly noted changes in the Damariscotta, while warmer winters and coastal development are important to participants in the Medomak (Fig. 5). We also categorized hypotheses about the observed drivers of specific changes in the focal SESs (Table 3).

### Informing social–ecological systems research in coastal marine systems

Local knowledge maps of shellfish abundance and diversity, as well as information about system change from interview data, motivated the selection of long-term monitoring sites and led to the establishment of a community science project in the Damariscotta (Risley et al. 2023). Local knowledge data also identified locally relevant concerns and questions that can inform future research hypotheses. For example, participants observed increases in aquaculture activity and the emergence and rise of the wild oyster fishery (Fig. 3; Table 4). Participants posited that wild oyster populations have increased because oyster aquaculture provides a larval supply for wild populations (*n* = 3) (Table 4). Pelagic larvae from oysters raised on subtidal aquaculture farms reach the intertidal zone and recruit as wild oysters. A Damariscotta participant explained:

“When I started there were not many oysters to be found on the rocks, the ‘wild oysters.’ I think the general conclusion and assumption is that the oysters that are now showing up on the rocks are spawned from the farmed population.”

**Table 4:** Changes observed by participants in the Damariscotta and Medomak focal SESs (total *n* = 44) and hypotheses shared by participants about the drivers of these changes, as categorized by the research team. Because participants often provided multiple drivers for each observation, the sum of n-values for drivers may exceed those of observed changes. ‘Aggregated’ refers to cases where participant observations were pooled as they were mentioned by fewer than 3 participants

Observed changes	*n*	Hypothesized drivers of change	*n*
Soft-shell clam populations have declined	13	Mud is not aerated by digging/harvesting activity	3
		Overharvesting	3
		Green crab predation	3
		Aggregated: predation of adult and larval clams by milky ribbon worms, birds, farmed oysters, and other species	6
Aquaculture activity has increased	10	Aquaculture is a good professional/economic opportunity	4
		Aquaculture technology has improved	3
		The Damariscotta has ideal aquaculture conditions	3
		Local research and educational organizations have supported aquaculture training and research	3
		Market demand for oysters has increased	2
Recreational boating activity and kayaking has increased	9	Human population growth	3
		Tourism activity has increased	2
The shape and composition of the estuary and its shores are changing	8	Climate change/sea level rise	3
		Dragging of the benthic environment to harvest farmed oysters	2
Soft-shell clam populations fluctuate and are hard to predict	6	Natural cycles/nature	4
		Overharvesting	2
Development has increased	5	Human population growth	3
Mussel populations have declined/disappeared	5	Green crab predation	2
The number of shellfish harvesters has declined	5	Water quality has declined and increased flat closures, limiting access to the shellfish resource	2
		There are a limited number of licenses/ difficult to get licenses	2
		Social and cultural changes	2
Tourism activity has increased	5	Aquaculture industry growth (the focus of tourism)	3
		COVID-19 pandemic	2
Green crab populations fluctuate with temperature	5	Warm temperatures support green crab growth and development	5
Green crab populations have increased	4	Climate change/warming seasons contribute to conditions that are more favorable to green crabs	2
River access has decreased	4	Increased use, congestion at local boat launches	3
River navigability has declined	4	Aquaculture gear, like floating cages (affect navigation)	4
Water quality flat closures have increased	4	Aggregated: continued poor water quality, combined with more extreme weather events and runoff	4
Aquaculture is negatively impacting soft-shell clam populations	3	Aquaculture bottom dragging suspends sediments that smother clams	3
Conservation closures are good for shellfish populations	3	Closures allow for stock regrowth	3
Eelgrass habitat has declined	3	Green crabs dig out eelgrass	2
Mussel distributions have shifted from intertidal to subtidal areas (attached to ropes/lobster traps)	3	Aggregated: green crab predation, overharvesting, or natural cycles	3
Oyster populations negatively affect soft-shell clam populations	3	Competition for food among shellfish species	2
		Aggregated: wild oysters eat clam spat or sedimentation from aquaculture dragging	2
Quahog populations have increased	3	Aggregated: climate change/warming waters, resistance to predation pressure, or benefits of living near the surface	3
Soft-shell clam intertidal distribution has changed from fine sediments to firmer sediments	3	Aggregated: fine sediments have more predators, the shells in the firm sediments buffer water acidity, or harvesters like to dig the soft mud, and these areas get dug out first	3
Wild oyster populations have developed and increased	3	Larvae from farmed oysters has led to the emergence of wild oyster populations	3

Another participant described this phenomenon in detail:

“There’s millions of oysters being grown on [the Damariscotta’s aquaculture] farms. But they’re still wild animals and they still go through a reproductive phase. And when they do that, the oyster is a free-swimming larva for two or three weeks. And it may or may not land on the farm. And could just equally land on the rocks along the shore and into the intertidal zone… I see wild oysters all over the place–they are now coming into their own and will start to self-develop. But it really was predicated by the oyster industry.”

The appearance of wild oysters and the concurrent decline of soft-shell clams has changed the composition of shellfish populations in the Damariscotta and altered how commercial harvesters perceive and use the estuary. A participant from the neighboring Medomak observed:

“…in the past 30 years oysters and oyster farming has taken off in Damariscotta, it’s nothing but oyster farming on the river right now. And it’s solid full of natural oysters because of the overflow from the farms.”

**Fig. 6:**
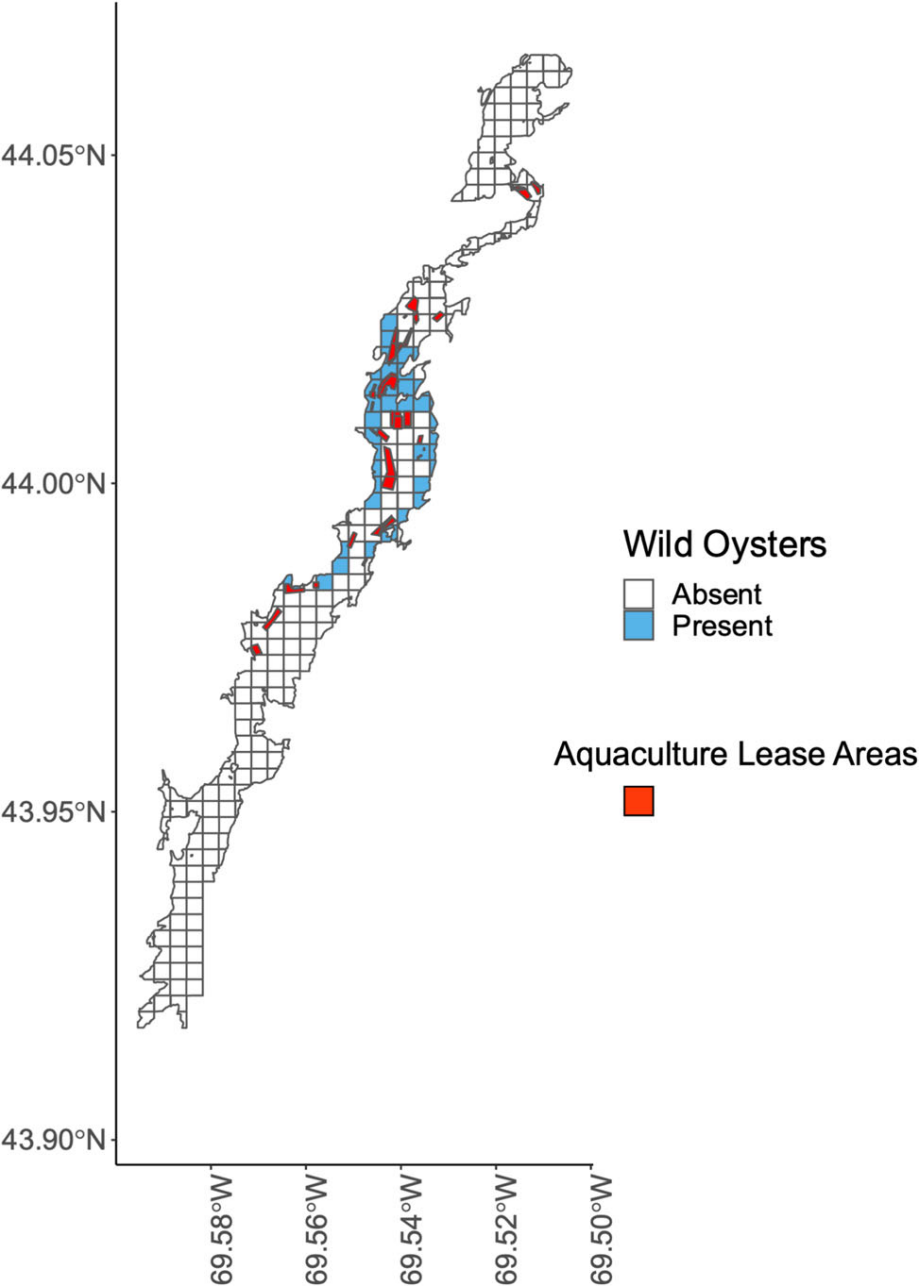
Distribution of wild oyster populations and aquaculture leases in the Damariscotta River (*n* = 10). Aquaculture lease sites are from the Maine Department of Marine Resources Aquaculture Lease Map for 2021, when the participatory mapping study occurred

These data indicate spatial overlap between locations of oyster aquaculture and the distribution of wild oyster populations. Figure 6 synthesizes shellfish harvesters’ observations of wild oysters in the Damariscotta and how these observations relate to the boundaries of current aquaculture farms, as represented by lease area, in the upper Damariscotta River.

Adding further to the context of changing shellfish populations and increased aquaculture activity, participants expressed their beliefs that aquaculture activities could negatively affect soft-shell clam populations. Participants cited bottom dragging—the practice of pulling a drag behind a boat to retrieve oysters raised on the seafloor—and competition for food between oyster populations (both wild and farmed) and soft-shell clams (*n* = 2; *n* = 2) (Table 4). Participants explained that they felt that dragging can “silt out” the mudflats: that is, the sediment suspended in the water column by the dragging activity could smother adult and juvenile soft-shell clams. Regarding competition among oysters and soft-shell clams, participants pointed to the oyster, with its high filtration capacity, as a potential predator of soft-shell clam larvae: “Basically like a whale that eats the plankton…when the clams lay their spawns, they float, and the oysters siphon them in.” Participants also shared observations of public perceptions and potential conflict, specifically in the Damariscotta:

“It seems like the conclusion is that those ‘damn oyster farms’ are sucking up all the clam larvae or they’re taking all the feed…Personally, I’m not convinced at all that that’s true And I think that…the recruitment boxes [show] pretty clearly that there are places that have plenty of larvae. But those larvae just don’t survive post settlement because the predation is too high. So, I suspect it’s much more of a predation problem than oyster farms killing off the feed or killing off the larvae problem.”

Interview data also revealed that shellfish harvesters are wary of welcoming shellfish aquaculture into their home SESs. As expressed by a Medomak participant regarding how harvesters address any oysters that they encounter within the estuary: “Everybody [who] finds oysters…takes them home, gets rid of them. We want to stay soft-shell clam because of what we are and who we are and where we are. And river aquaculture is frowned on in Waldoboro…none of that’s welcome. We are wild clammers and we want to stay that way.”

## Discussion

Local knowledge provides valuable information to understand social and ecological change and guide the design and development of collaborative research and ecosystem stewardship in coastal marine SESs. By grounding research in local knowledge and participatory approaches, researchers can better support community-based decision-making with place-based, fine-scale information, while simultaneously cultivating collaborative relationships with community members and other partners.

The research presented here was foundational for the development of a collaborative community science project in the Damariscotta River estuary focal system (Risley et al. 2023). This project initially emerged from requests from the Damariscotta-Newcastle Joint Shellfish Conservation Committee and the Bremen Shellfish Conservation Committee to learn more about how and why shellfish species populations are changing. The results of the participatory mapping study generated testable hypotheses and actionable information that have since informed collection of additional social and ecological data examining the interplay among social and ecological processes in the study system. For example, observations and hypotheses regarding the social and ecological connections between oyster aquaculture and the shellfish fishery informed the community science project research design by: (1) identifying key species interactions to investigate (e.g., oyster and soft-shell clam populations—Table 4), (2) narrowing the geographic scope of the study area (e.g., areas of overlap between aquaculture and wild oyster populations—Fig. 4), and (3) highlighting areas of complexity or potential conflict (e.g., harvester fears concerning the potential negative impacts of aquaculture). Ultimately, participatory mapping of local knowledge can inform the “where, how, what, and why?” of collaborative research projects.

### Spatial and temporal information to frame understandings of change

Local knowledge maps like those produced from this study provide spatially explicit information guiding the "where" of collaborative research. In the short-term, maps can inform decisions about optimal areas for conservation or restoration. They also guide long-term monitoring and other spatial research. Maps highlight locations of importance, either for their economic significance as primary harvesting grounds with high soft-shell clam abundance (Fig. 3) or for their ecological significance as locations of high shellfish diversity (Fig. 4). The local knowledge shared during our study informed the selection of long-term monitoring sites and raised important questions about locations of overlapping social and ecological uses and processes. For example, our maps highlighted the spatial overlap between oyster aquaculture and wild oyster populations (Fig. 6). In response, investigating this spatial interaction became a primary focus of the collaborative research in the Damariscotta by our research group.

Likewise, local knowledge, a system of knowledge that typically spans multiple seasons and decades, is key to understanding long-term changes over large spatial and temporal scales, contextual information that informs the "how" of collaborative research (Moller et al. 2004). Local knowledge can aid in interpretations of fine-scale changes and patterns through time. Particularly in systems where data are limited, and extensive data collection is not possible, local knowledge enhances access to long-term social and ecological data to guide more targeted approaches to research and management (e.g., Loch and Riechers 2021). Significantly, this study enabled harvesters to share their observations and hypotheses about why shellfish populations are changing, informing future research avenues like the Damariscotta community science project and resource decisions (Table 4). These data document local knowledge holders’ observations of ecological change, like the decline of soft-shell clam populations, paired with hypothesized drivers of the change in question, including overharvesting or predation by the invasive European green crab (*Carcinus maenas*), a voracious soft-shell clam predator (Tan and Beal 2015).

Local knowledge also identifies locally specific social and ecological factors that guide the "what" of collaborative research. The list of local knowledge participant observations and hypothesized drivers, which consist of both social and ecological dimensions, directly informed the design of our collaborative research project. For example, we designed a multispecies survey methodology for the Damariscotta community science project that spans both soft sediment and hard substrate shellfish species habitats, based on participant observations that identified these as variables in need of investigation (Table 4; Fig. 4) (Risley et al. 2022). We also included a protocol to study green crab population abundance (McMahan 2020) at each shellfish monitoring site, and we selected monitoring sites with a range of harvesting activity (i.e., open to harvest year-round, closed to harvest seasonally, and closed year-round) to investigate this factor as a hypothesized driver of change. Building collaborative research on local knowledge informed where research should occur and what variables should be investigated.

### Local knowledge frames the "why" in collaborative social–ecological research

The process we present here, in which research begins with local knowledge and participatory methods, is an example of how collaborative and iterative methods can lead to more ethical and constructive engagements between researchers and coastal communities. Our findings illustrate how participatory methods provide opportunities where local knowledge and conventional science can be woven together with other knowledge systems.

In this study, we found that local knowledge is uniquely suited to capturing ecological and social data in tandem and as a result is an ideal approach for informing social–ecological research scope and design in complex coastal SESs. Local leaders are tasked with decision-making related to environmental and social changes and require local-level information to guide decisions. Understanding the social and ecological dynamics at play in a system can not only guide study design but also provides richer context for the motivations behind various approaches to research. We found this through our research, where local knowledge refined the focus on multiple shellfish species and their predators and highlighted key hypotheses, such as the interactions between wild shellfish and aquaculture, to guide future collaborative research. The scale and scope of understanding a landscape, whether ecologically or socially, require extensive time, resources, and effort often unavailable to communities and researchers. For shellfish managers, who frequently lack the technical and financial means for research and monitoring, local knowledge has the capacity to provide new social and ecological data and generate collaborative options for future research (Lima et al. 2017).

Research approaches like participatory mapping help to enhance relationships among researchers and coastal communities due to its collaborative nature and their mobilization of diverse bodies of knowledge (Kobluk et al. 2021). The relationships we developed with participants over the course of the study formed the foundation for a continued collaborative relationship with harvesters and shellfish resource managers in the focal SESs. Beginning with local knowledge establishes an expectation and example of knowledge co-production that can inform the structure and design of future research initiatives. Through the local knowledge documentation process, researchers listen and learn, analyze results, and share these findings back with community members to solicit feedback. In this case, we shared the results of this study at multiple community meetings and met with shellfish managers throughout the collaborative research design process. Such early and ongoing communication has proved essential for collaborative research projects (Pearce et al. 2009). Collaborative research projects that result from the local knowledge process help to build trust and mutual understanding among resource users, managers, and researchers (Lebel et al. 2006). Trust allows for dialogue around appropriate data collection and management techniques. As a result, coastal communities are more able to participate in stewardship actions due to better data, more credible methods, and established avenues to disseminate information.

Weaving together diverse forms of knowledge can be challenging, but common ground can be negotiated and valuable insights can be gained as part of the collaborative research process (Ruddle and Davis 2013). Such efforts can advance understanding and management in coastal SESs by providing timely, locally relevant information grounded in real-life experience (St. Martin et al. 2007).

### Remaining challenges and considerations

Local knowledge data are complex and heterogeneous. At times, local knowledge contains contradictions—as conventional scientific research does, argued Dey et al. (2020). It often is difficult to standardize according to scales of time, spatial coverage, and expertise (St. Martin et al. 2007; Dey et al. 2020). Nonetheless, as our study shows, it is a rich body of knowledge that can catalyze further knowledge generation and collaboration. Coastal research and stewardship should explore more ways to engage with local knowledge to support resilient and adaptive research and stewardship in coastal marine SESs.

Despite recent progress in collaborative research, local knowledge is often treated as something of lesser value than knowledge generated or curated by professional researchers. Researchers and resource managers continue to struggle to fully connect local knowledge with scientific information (Hind 2015). When local knowledge is "integrated" with western scientific knowledge, it is often treated as just another form of data, subject to the structures and power hierarchies implicit within western concepts of science and "knowledge." Local knowledge is not simply a new form of "data" to be integrated into scientific research and researchers should be careful to not concentrate power away from local knowledge holders and resource users (Berkes 2009; Stephenson et al. 2016). If founded in collaborative efforts that account for power dynamics among fishers, scientists, and other stakeholders, local and conventional scientific knowledge can interact in ways that enrich both knowledge cultures.

Our study offers one example of how participatory mapping of local knowledge can be valuable for informing collaborative research and management in marine coastal SESs. But local knowledge has its limitations. That is, local knowledge is often derived from individuals’ experience and personal observations, potentially leading to biases when understanding social and ecological change (Bessesen and González-Suárez 2021). Additionally, local knowledge is place based. While this is beneficial for some projects, local knowledge may not fully capture broader ecological trends, especially in the face of climate change, which impacts species distributions and ecosystem dynamics beyond the scope of local insights (Johannes et al. 2000). The transmission of local knowledge across generations may also be declining, e.g., as youth leave rural communities; this can reduce the continuity of local knowledge contributions (Brown and Kyttä 2018; Johnson 2020). Addressing these concerns requires developing collaborative frameworks that respect and validate local knowledge while ensuring it complements other forms of knowledge for sustainable marine ecosystem management.

Ultimately, our study makes several contributions to ongoing research of how local knowledge helps to adapt collaborative research projects to the unique needs and priorities of coastal marine SESs. This foundational research and the resulting collaborative community science project (Risley et al. 2023) demonstrate that participatory mapping informs collaborative research in many ways, including: (1) identifying locations of social and/or ecological importance for monitoring or inquiry; (2) informing the type of data researchers collect in future studies, based on relevant questions and needs; (3) developing testable hypotheses and actionable information at a scale relevant for stewardship of coastal SESs and (4) fostering partnerships, stakeholder engagement, and thinking at the level of the social–ecological system before on-the-ground research begins. While this work is ongoing, local knowledge and collaborative research approaches, like the mapping applied in this study, are clear steps forward in the process of ecosystem stewardship. Continued efforts to sustain the dialogue between social and ecological research, particularly that focused on local knowledge, will build capacity for systems-level thinking and move us closer to proactive, locally relevant, and inclusive ecosystem stewardship in complex coastal marine SESs. This paper advances SES research by demonstrating a method of research and knowledge generation in coastal marine SESs that can connect complex outcomes with possible social and/or ecological variables. Drawing on the fields of ecology, geography, anthropology, and sociology, this research is important for demonstrating interdisciplinary, participatory research in complex SESs. Methods that document these associations and recognize the multi-causal nature of outcomes can be the first step in the systematic study of governance in coastal marine SESs (Basurto et al. 2013).

## Supplementary Information

Below is the link to the electronic supplementary material.

Supplementary file1 (PDF 717 KB)
