# Supplementary material for: Mapping local knowledge supports science and stewardship
Source: Ambio. 2025 Apr 26;54(10):1648–65. doi: 10.1007/s13280-025-02170-4 (PMC12405077; doi:10.1007/s13280-025-02170-4)
Supplement: Supplementary file 1 — Supplementary file1 (PDF 717 KB) [file 13280_2025_2170_MOESM1_ESM.pdf]

CODING SCHEMA & DEFINITIONS

Supplementary Table 1: Semi-structured interview coding schema. Schema includes top level themes (bold), secondary themes (italic), and secondary theme definitions (plain text).

**Access**								
Access: Physical	*Access: Financial*	*Access: Legal*						
Participant refers to access to a physical place, such as the town boat ramp or shoreline. Also includes mentions of where a participant accesses the river from.	Participant describes limited access due to financial barriers. For example, limited access to certain fisheries due to the cost of the license.	Participant discusses legal restrictions that limit access. For example, the move from state to town licenses that limited access to only town of residence mud flats.						
**Change**								
Change: Species Change	*Change: Spatially Widespread*	*Change: Spatially Isolated*	*Change: Physical habitat*	*Change: Access*	*Change: Demographic*	*Change: Uses*	*Change: Aquaculture*	*Change: Economic*
Participants mention changes in the abundance and distribution of species	Participants describe a change that occurred over the entire river system (sub-codes for gradual vs. rapid change).	Participants describe a change that occurred over a small area of the river (sub-codes for gradual vs. rapid change).	Participants describe changes that explicitly reference the physical habitats of the river, like sediment, temperature, salinity, depth, etc.	Participants mention changes in how they access the water.	Harvester changes, property ownership, general demographics, loss of young people.	Kayaking, paddleboards, boating, sailing (Decrease)	Sub-codes: Aquaculture growth, Aquaculture ecosystem effects.	Like changes in fisheries or increases in tourism. Discussions about the opportunities that aquaculture provides will be coded separately in "Economic Opportunity"

Supplementary Table 1 continued.

Climate Change								
Climate Change: Sea level rise	*Climate Change: Water temperature*	*Climate: Changes in Seasons*						
Participant discusses observations of sea level rise in the river.	Participant refers to warming water temperature and its possible effects.	Participant references milder winters, longer summers, and the possible effects.						
**Conflict**								
Conflict: Crowding	*Conflict: Tourism*	*Conflict: Shore Use*	*Conflict: Worm harvesting industry*					
Participant discusses conflict related to crowding in the river. This includes boat activity (kayakers vs. powerboats, sailing vs. aquaculture lease sites) in the open water, or crowding in important public spaces like boat launches.	Participant describes conflict related to tourist activity in the river. This includes issues related to increased noise pollution due to tourist activity or concerns related to inexperienced tourist boaters.	Participant describes conflict related to accessing or using the shore or intertidal zone. This most often relates to access to the shore for shellfish harvesting activity.	Participant describes conflict related to marine worm harvesting activity.					
**Demographics**								
Age	*Gender*	*House Location*	*Occupation*	*Use Frequency*	*Use Location*	*Use Season*	*Use Years*	
Age of participant.	Gender of participant.	Location of current home.	How the participant uses the river. Can be occupation or other experience using the river.	Number of times per week/month/year that participant is on the river.	Locations in the river used most frequently by the participant.	Season of use/activity on the river by the participant.	Number of years the participant has used the river.	

Supplementary Table 1 continued.

Development & Growth								
Development: House-Dock Construction	*Development: Long-Term Population Growth*	*Development: COVID-19 Growth*	*Economic Opportunity*					
Participant describes the building of new homes or infrastructure on the river. For example, the building of docks and the development of previously uninhabited land.	Participant discusses long-term trends in population growth. For example, the popularity of Damariscotta as a retirement location and the subsequent increase in seasonal or year-round residents.	Participant discusses the short-term population growth as a result of the COVID-19 pandemic. For example, increased housing prices as a result of more people permanently moving to Maine to get away from big cities.	Participant discusses economic opportunities related to the river systems. Due to aquaculture, growth of tourism, or the return of commercially important marine species.					
**Hypothesis**								
Hypothesis: Hypotheses	*Hypothesis: Drivers*	*Hypothesis: Hyps about the resource*						
**The hypotheses are any description of change that has a clear driver, so the participant articulates why a change has happened. This node is developing. For now, we're coding all changes to this node and will subdivide if needed. We'll code positive and negative interactions later*	Within the coded hypothesis, code the thing causing the change. This may need to be further subdivided?	This can also include ideas from harvesters about why clams declined in the first place and what they think needs to be done (like turning the mud regularly) to improve the health of the clam resource.						

Supplementary Table 1 continued.

**Local Knowledge (LK) Ecological Understandings**								
LK: Complexity		*LK: Cycles*						
When participants are talking about environmental processes, they refer to the complexity of marine ecosystems - this is usually a comment in reference to the difficulty in predicting environmental change and/or the difficulty of understanding why things have changed. They refer to "mother nature" and trust in nature to find a balance and recover from disturbance.		When participants are talking about environmental processes, they refer to nature as cyclical. They might seem unconcerned about current declines because they have seen increases and declines in the past. This might also be a comment about the difficulty of making predictions and understanding change						
**Management (Mgmt)**								
Mgmt: State	*Mgmtt: Town Licenses*	*Mgmt: Conservation Efforts*	*Mgmt: Uncertainty*	*Mgmt: Power*	*Mgmt: Illegal Harvest*			

Participant describes management of the rivers/species and refers to state-level agencies like DMR	Participants describe management rivers/species and refer to town-level management, including shellfish and harbor committees. This will likely mostly involve the management of shellfish. *Updated to include general comments about town licenses that don't mention access changes	Participants describe things that are or could be done to contribute to shellfish management. This includes shellfish committee conservation work and proposed alternative methods, like closures or brushing.	Participants mention difficulties navigating the management system, either for themselves or others. This could include uncertainty about how the management system works or observed uncertainty of other people.	Participant mentions power imbalances between harvesters/users and management agencies (either state or town).	Participant mentions 'illegal' or unpermitted/unlicensed harvest of species from the river.			

Supplementary Table 1 continued.

**Power**								
Power: Hierarchy among river user groups								
Participant refers to hierarchy or class structures/economic inequality that influence access, control, or decision-making in the river system.								
**River Activities**								
Activity Seasonality	*Activity Intensity*	*Activity Location*	*Aquaculture*	*Commercial Fishing*	*Other*	*Rec Boating*	*Rec Fishing*	*Research*

Participant describes the time year when certain river activities take place.	Participant describes the density and intensity of activities in different parts of the river. For example, the participant mentions an increase in kayaking or an area of high boat traffic.	Location of activities, including shellfish harvesting.	Aquaculture	Sub-codes: Elvering, Lobstering, Other Commercial Fishing, Shellfishing		Sub-codes: Kayaking, Motorboating, Other Boating, Sailing		Participant describes research activities that they have observed, lead, participated in, or heard about on the river.
**River History**								
River History								
Participant mentions activities that took place on the river or species that were present >100 years ago								

Supplementary Table 1 continued.

**Sediment**								
Sediment: Erosion	*Sediment: Suspension*	*Sediment: Restructuring*						

Participant describes shore loss due to erosion.	Participant discusses suspended sediment in the water column and its possible effects. For example, suspended sediment due to bottom aquaculture dragging.	Participant describes changes in the channel shape, sand bars, or other structures in the river due to the movement of sediment.						
**Shellfish**								
Shellfish Habitat	*Shellfish Distribution*	*Shellfish Abundance*	*Public Health*	*Predators*				
Participant describes the shellfish habitat for specific species. This includes the physical environment description, including substrate type. Includes wild oysters.	Participant describes the physical distribution of the shellfish. This includes tidal height or depth in sediment, as well as seasonal and temporal variations in distribution. Includes wild oysters.	Participant describes the abundance of shellfish species. This can either be spatial (i.e., There are a lot of clams here...) or temporal (i.e., The number of clams has declined in the last 15 years).	Participant mentions public health concerns related to shellfish. This can include references to vibrio, flat closures due to bacteria, or poor harvesting practices that could cause public health concerns.	Participant mentions predators that affect shellfish species, such as green crabs or milky ribbon worms.				

Supplementary Table 1 continued.

**Shellfish Fishery**

SSClam: Decreasing #/ aging harvester pop	*SSClam: Percent of total income*	*SSClam: Harvesting methods, strategies and species*	*SSClam: Harvesting locations*	*SSClam: Threats to the fishery*	*SSClam: Methods to improve the fishery*	*SSClam: Views of occupation*	*Harvest Rate*	
Participant discusses demographic changes or changes in the total number of shellfish harvesters. Participants refer to harvesters 'aging out' or retiring, as well as a lack of young harvesters entering the fishery. Subsets include decreasing harvesters due to 1) Harvester aging and retirement and 2) Limited entry of young people to the fishery due to how labor intensive and seasonal nature of the work.	Participant discusses financial or circumstantial aspects of the work (more of a part-time/seasonal job, difficulty to make it year-round work due to town restrictions).	Participant describes which species they harvest and how. For example, 'picking' for oysters and digging for clams, or harvesting a suite of different species so that they can respond to changes in market prices.	A means to capture the important harvesting sites in both river systems. To track the names and locations of important coves, or cove of historical importance.	Includes flat closures due to pollution, overharvesting, too many harvesters, licenses, aquaculture harming clam pops/settlement, or aquaculture industry threatening livelihood of clammers.	This can also include ideas from harvesters about why clams declined in the first place and what they think needs to be done (like turning the mud regularly) to improve the health of the clam resource.	Participant refers to shellfishing as work similar to farming--the mudflat as a garden, or other agricultural metaphors.	Participant describes the quantity of shellfish harvested in a tide/day/etc.	

Supplementary Table 1 continued.

**Tourism**

Tourism: Increased activity	*Tourism: Inexperience*	*Tourism: Aquaculture-related*	*Tourism: Increased activity due to COVID-19*	*Tourism: Development*				
Participant mentions increases in tourism activity on the river but not with respect to COVID-19?	Participants mention tourists who are inexperienced being on the water and put themselves in danger, either by illegally gathering shellfish or kayaking where they shouldn't.	Participant links tourism to the aquaculture industry on the DRE. This could be general, like how oysters are cool now and people want to learn about real-life farmers, or it could be one of many mentions of the red tour boat.	This refers to an observed (or not) spike in river tourism in 2020 and linked to COVID-19.	This refers to property conversions (either seasonal or short-term rentals) and longer-term demographic changes in the area that are driven by tourists.				
**Water Quality**								
Water Quality: Pollution	*Water Quality: Improvement*							
Participants mention water pollution and declines in water quality. *Do we care about the type of pollution mentioned? And the cause.	Participants mention improvements in water quality in an area. Capture where this has occurred.							

Supplementary Table 1 continued.

Inductive								
Inductive: General Map Feedback	*Inductive: What we Missed*	*Other*	*Interesting things to think about*	*In-Vivo*	*River Species: In Vivo*			
Participant comments about the map.	Things the participants identified that we missed in this study.	This node developing, MLB and SCR check in periodically and adjust if needed.	Open-ended: MLB and SCR tag interesting ideas that aren't necessarily relevant to coding but might be useful.	Open-ended: MLB and SCR using this to grab quotes that might be useful later	We code the first time a participant mentions each species.			
